# Interactions between Germline and Somatic Mutated Genes in Aggressive Prostate Cancer

**DOI:** 10.1155/2019/4047680

**Published:** 2019-03-17

**Authors:** Tarun Karthik Kumar Mamidi, Jiande Wu, Chindo Hicks

**Affiliations:** Department of Genetics, Louisiana State University Health Sciences Center, School of Medicine, 533 Bolivar St., New Orleans, LA 70112, USA

## Abstract

Prostate cancer (PCa) is the most common diagnosed malignancy and the second leading cause of cancer-related deaths among men in the USA. Advances in high-throughput genotyping and next generation sequencing technologies have enabled discovery of germline genetic susceptibility variants and somatic mutations acquired during tumor formation. Emerging evidence indicates that germline variations may interact with somatic events in carcinogenesis. However, the possible oncogenic interactions and cooperation between germline and somatic variation and their role in aggressive PCa remain largely unexplored. Here we investigated the possible oncogenic interactions and cooperation between genes containing germline variation from genome-wide association studies (GWAS) and genes containing somatic mutations from tumor genomes of 305 men with aggressive tumors and 52 control samples from The Cancer Genome Atlas (TCGA). Network and pathway analysis were performed to identify molecular networks and biological pathways enriched for germline and somatic mutations. The analysis revealed 90 functionally related genes containing both germline and somatic mutations. Transcriptome analysis revealed a 61-gene signature containing both germline and somatic mutations. Network analysis revealed molecular networks of functionally related genes and biological pathways including P53, STAT3, NKX3-1, KLK3, and Androgen receptor signaling pathways enriched for germline and somatic mutations. The results show that integrative analysis is a powerful approach to uncovering the possible oncogenic interactions and cooperation between germline and somatic mutations and understanding the broader biological context in which they operate in aggressive PCa.

## 1. Introduction

Despite remarkable progress in patient screening using the prostate specific antigen (PSA) and improved patient care, prostate cancer (PCa) remains a major public health problem [1, 2]. PCa is the most diagnosed cancer and the second leading cause of cancer-related death among men in the US [1, 2]. The majority of patients present with localized tumors that will remain indolent and pose no harm even without treatment [3]. However, a significant proportion of patients ~ 20% to 30% will develop aggressive tumors, which progress rapidly to metastatic form and is often lethal if not detected and treated early [3, 4]. Therefore, there is an urgent need for the discovery of molecular markers for early detection of aggressive PCa.

Advances in high-throughput genotyping and reduction in genotyping costs have enabled discovery of genetic variants, primarily single nucleotide polymorphisms (SNPs) herein called germline mutations associated with an increased risk of developing PCa using genome-wide association studies GWAS [5]. These findings are providing foundational knowledge about the genetic susceptibility landscape of PCa. Most notably, these discoveries are being incorporated into risk prediction models for identification of patients at high risk of developing aggressive disease [6, 7]. For example, recently, Seibert et al. developed and validated a polygenic hazard score to guide screening for aggressive PCa [7]. However, despite this remarkable success, establishing the link between genetic predisposition and tumorigenesis remains a challenge. This knowledge gap has hampered translation of genetic susceptibility variants from GWAS studies into clinical practice to improve human health.

The recent surge of next generation sequencing of the cancer genomes has led to an expanded molecular classification of PCa and discovery of somatic driver mutations acquired during tumorigenesis [8]. Large multicenter projects such as The Cancer Genome Atlas (TCGA) [9] and the International Cancer Genome Consortium (ICGC) [10] have performed a series of detailed analyses of the somatic alterations affecting tumor genomes in sporadic PCa. Discoveries from these large-scale studies are providing valuable information about the genomic landscape of the tumor genomes. However, to date, somatic mutation information has not been maximally leveraged and integrated with information on germline genetic susceptibility variants to infer the possible oncogenic interactions and mechanisms of cooperation between germline and somatic variation in aggressive PCa. Emerging evidence indicates that germline variations may interact with somatic events in carcinogenesis [11, 12]. Recently Wang et al. reported interactions between germline genetic susceptibility loci and somatic alterations in lung cancer [11]. Feigelson et al. proposed approaches for integrating germline and tumor genomic data in cancer research [12]. However, the possible oncogenic interactions between germline and somatic alterations in aggressive PCa have not been reported. The objective of this exploratory study was to investigate the possible oncogenic interactions and cooperation among and between genes containing germline mutations and genes containing somatic mutations in aggressive PCa. Our working hypothesis was that genes containing germline and somatic mutations are functionally related and interact in gene regulatory networks and biological pathways driving aggressive PCa. To address this hypothesis we combined germline mutation information derived from GWAS with somatic mutation information from TCGA using gene expression data on aggressive PCa from the TCGA. Our evaluation focuses on genes containing germline and somatic mutated rather than individual mutations to understand the broader biological context in which they cooperate and to establish putative functional bridges between germline-somatic interactions and the pathways they control.

## 2. Materials and Methods

### 2.1. Germline Mutations and Associated Genes

We have developed and published a comprehensive catalogue of genetic susceptibility variants (SNPs) and genes reported to be associated with an increased risk of developing PCa from GWAS [5]. For this study, we used an updated version of this catalogue. The information in this catalogue was supplemented with information from the GWAS catalogue [13] to ensure completeness of the information used in this study. Details about methods used in collection of genetic susceptibility variants and genes from GWAS have been described in our earlier publication [5]. The methods for data collection were based on the guidelines proposed by the Human Genome Epidemiology Network for systematic review of genetic associations [14–18], which we have described elsewhere [5]. Here, we provide a brief, but detailed description of the data used in this study as well as the inclusion and exclusion criteria used.

We reviewed over 200 published reports on GWAS on PCa. The reports were screened by title, abstract, and full-text review to identify the studies meeting our eligibility criteria. Because most of the GWAS reports have not been clinical phenotype-specific (i.e., indolent or aggressive PCa specific), we reviewed all GWAS studies available on PCa. After screening, 150 studies that met our eligibility criteria were selected and subjected to further detailed review. The exclusion criteria for the 50 studies included removal of studies with insufficient or incomplete information, reviews, studies reporting only intergenic regions, and studies with very small sample sizes (i.e., studies containing <500 subjects in cases and controls). For the remaining 150 studies used in this study, they were considered eligible if they met the following criteria: (1) the studies must have been based on a case-control study design using unrelated individuals, (2) publications must have been of full length and published in peer-reviewed journals or online in English language before June 2018, (3) PCa must have been diagnosed by histological examination, (4) the sample sizes must be more than 500 for the cases and more than 500 for the controls to reduce sampling errors, (5) the study must have provided sufficient information such that genotype frequencies for both PCa and controls can be discerned without ambiguity, and (6) the studies must have used the appropriate and recommended statistical methods to infer the associations by taking into account the covariates and accounting for population structure and genetic background [14].

We manually extracted the information from the 150 studies meeting our eligibility criteria and the accompanying Web sites containing supplementary data. The extracted information included SNP identification number (rs-ID); evidence of association as determined by the GWAS* P *value; a composite of strong (*P *⩽ 10^−8^), moderate (*P* = 10^−5^–10^−7^), and weak (*P* = 10^−2^–10^−4^) association; gene name; and associated chromosome position to which the genes map as determined by the dbSNP database [19] and the Human Genome Nomenclature database [20]. This search yielded more than 500 SNPs in introns and exons mapped to 266 genes from a population of more than 250,000 cases and more than 250,000 controls. The GWAS data set included 61 genes containing genetic variants directly associated with an increased risk of developing aggressive PCa. The database was cross-referenced with the GWAS catalogue database for validation and to ensure that all reported genetic variants and associated genes are represented in the data set. A complete list of genetic variants and associated genes along with sources or published reports from which they were derived is presented in Table SA provided as supplementary data to this report.

### 2.2. Somatic Mutation Information and Gene Expression Data

Today, treatment decisions for PCa patients are guided by various stratification algorithms [21]. Among these parameters, the most potent predictor of PCa mortality is the Gleason grade which ranges from 6 to 10 in the modern era [21]. The presence of Gleason grade 6 is associated with very low cancer-specific mortality rates, even in the absence of intervention. Intermediate grade disease (Gleason grade 7) has a much more variable course. High Gleason grade 8-10 are aggressive and often lethal. For this study, we used somatic mutation information and associated genes derived from 188 patients with aggressive tumors (i.e., tumors with Gleason grad 8-10) from TCGA. In addition, because Gleason grade 7 follows a variable clinical course we used 4 + 3 score used by the American Urological Association to assign this group of patients to aggressive PCa [21]. For classification of tumors as aggressive, we used the clinical information provided by the TCGA and the classification protocols of the American Urological Association [21]. Somatic mutation information and genes were downloaded from the Genomics Data Commons and is available at https://gdc.cancer.gov/  [22].

Gene expression data derived from RNA-seq was downloaded from TCGA using Genomics Data Commons (GDC) data transfer tool along with clinical information at https://gdc.cancer.gov/  [22]. The gene expression data set included 305 patients with aggressive tumors and 52 normal control samples. Note that the number of patients with gene expression data is higher than the number of sequenced patients. The data matrix was filtered to remove rows with missing data, such that each row has at least ≥30% data using cpm (counts per million) filter (>0.5) implemented in R [23]. The resulting data set was normalized by TMM (The trimmed mean of M-values) normalization method and then transformed by Voom, using Limma package implemented in R [23]. The normalized data contained 18,428 probes and was used in the analysis. The probe IDs and gene symbols and names were matched for interpretation using the Ensemble database, a database used for gene annotation of sequencing experiments and sequencing technology platforms.

### 2.3. Data Analysis

**Figure 1:**
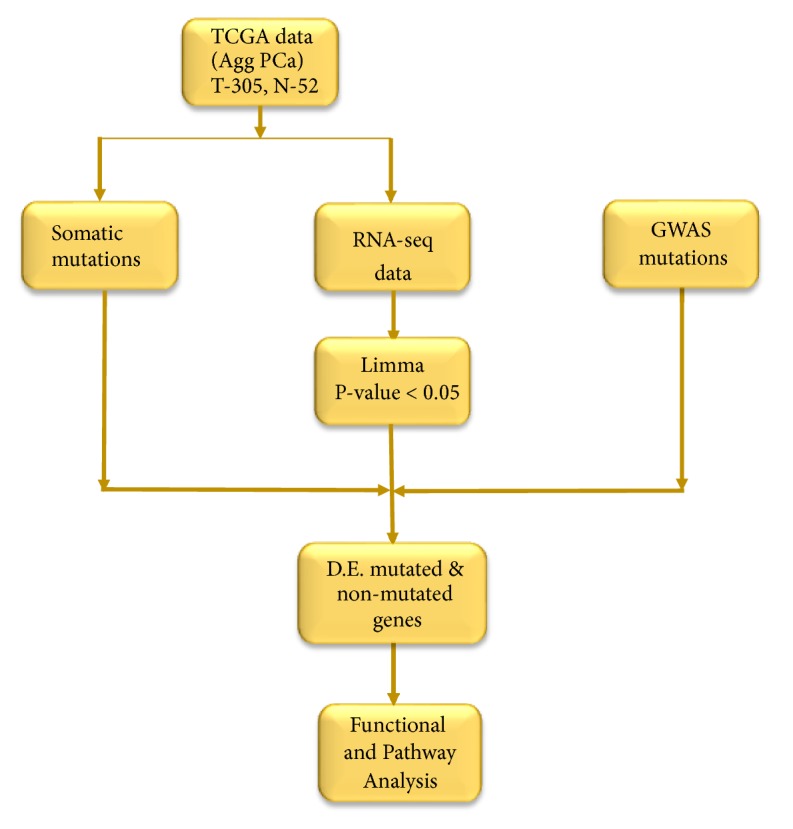
Design and workflow for integrative analysis combining germline with somatic mutation information using gene expression data. RNA-seq read count data and somatic information were downloaded from The Cancer Genome Atlas (TCGA) via the Genomics Data Commons. Germline mutation information was manually curated from GWAS studies. Limma (R) package was used for the discovery of differentially expressed (DE) mutated and nonmutated genes. Ingenuity Pathway Analysis (IPA) was used for functional analysis and discovery of molecular networks and biological pathways enriched for germline and somatic mutations.

The project design and data analysis workflow are presented in Figure 1. We performed unbiased whole genome analysis comparing gene expression levels between patients diagnosed with aggressive tumors and matched control samples using the Limma package implemented in R [23] to identify all significant differentially expressed genes distinguishing tumors from control samples. This unbiased approach was carried out to discover, germline and somatically mutated genes as well as nonmutated genes associated with PCa. We used the false discovery rate (FDR) procedure to correct for multiple hypothesis testing [24]. The genes were ranked on* P* values and the FDR. A gene was defined as PCa susceptibility gene if it belongs to protein-coding genes and contained the genetic susceptibility variant(s) and its expression in PCa tissue was associated with PCa risk SNPs. A gene was considered as PCa driver gene if it contained somatic mutations and there was evidence to be PCa related or the gene contained PCa-related driver mutations. A gene was considered to be involved in the germline and somatic genomes if it contained both germline and somatic mutations.

We performed hierarchical clustering using Morpheus [25] using sets of differentially expressed genes to identify clusters of coregulated genes and to assess similarity in patterns of gene expression profiles, among the germline and somatically mutated and nonmutated genes. Prior to clustering the data was standardized, normalized, and centered. For clustering, we used the Pearson correlation coefficient as the measure of distance between pairs of genes and the complete linkage as the clustering method. We performed enrichment analysis using Ingenuity Pathway Analysis (IPA) software [26] to identify molecular networks and biological pathways enriched for germline and somatic mutations. Using IPA, the most highly significantly differentially expressed genes distinguishing patients with tumors from control samples were mapped onto networks and canonical pathways. The probability scores and the log* P* values were calculated to assess the likelihood and reliability of correctly assigning the genes to the correct molecular networks and biological pathways. A false discovery rate was used to correct for multiple hypothesis testing in pathway analysis. The predicted molecular networks and biological pathways were ranked based on z-scores and log* P* values; respectively. Gene ontology (GO) [27] analysis as implemented in IPA was performed to characterize the functional relationships among genes in the networks as implemented in IPA. Genes were classified according to the molecular functions, biological processes and cellular components in which they are involved. Genes were considered interacting or cooperating if they were involved in the same molecular functions, biological process, cellular components, molecular networks, or biological pathways.

## 3. Results

### 3.1. Discovery of Somatic Mutated and Nonmutated Gene Signatures

To discover and characterize the somatic mutated and nonmutated gene signatures, we performed whole genome analysis comparing gene expression levels between patients with aggressive tumors and the controls samples. After controlling for multiple hypothesis testing, the analysis revealed two gene signatures. One gene signature consisted of 2,613 significantly (P<0.05) differentially expressed genes containing somatic mutations, of which 2,298 genes were highly significantly (P<0.01) differentially expressed. The other signature consisted of 10,192 significantly (P<0.05) differentially expressed genes containing no somatic mutations, of which 8,913 genes were highly significantly (P<0.01) differentially expressed. The results showing a signature of the top 34 most highly somatically mutated genes (≥ 5 mutation events) that were significantly differentially expressed are presented in Table 1. Also presented in the table are the number of somatic mutation events per gene and the differential expression* P* value.

The list of highly mutated genes included the genes* SYNE1, ATM, CTNNB1, FZD4,* and* RYR2* implicated in PCa [28–32]. A complete list of significantly differentially expressed somatic mutated and nonmutated genes including their mutation frequencies along with their estimates of* P* values and false discovery rates are presented in Supplementary Table S1A (somatic mutated) and Table S1B (nonsomatic mutated) provided as supplementary data.

### 3.2. Germline and Somatic Mutation Gene Signatures

To discover and characterize the germline and somatic mutation gene signatures, we evaluated the significantly differentially expressed genes between cases and controls. We evaluated the 266 genes containing germline mutations associated with an increased risk of developing PCa. To get a complete picture, we performed a series of investigations. First, we investigated whether genes containing germline variation also harbor somatic mutation and whether these genes are differentially expressed between patients with aggressive tumors and control samples. Specifically, we sought to discover a gene signature with germline and somatic mutations. Secondly, we investigated whether there are any genes containing germline variations that are differentially expressed between patients with aggressive PCa and control samples, but are not somatically altered. Thirdly, we investigated whether there are somatically altered genes that are differentially expressed between patients with aggressive PCa and control samples but are not altered in the germline.

**Figure 2:**
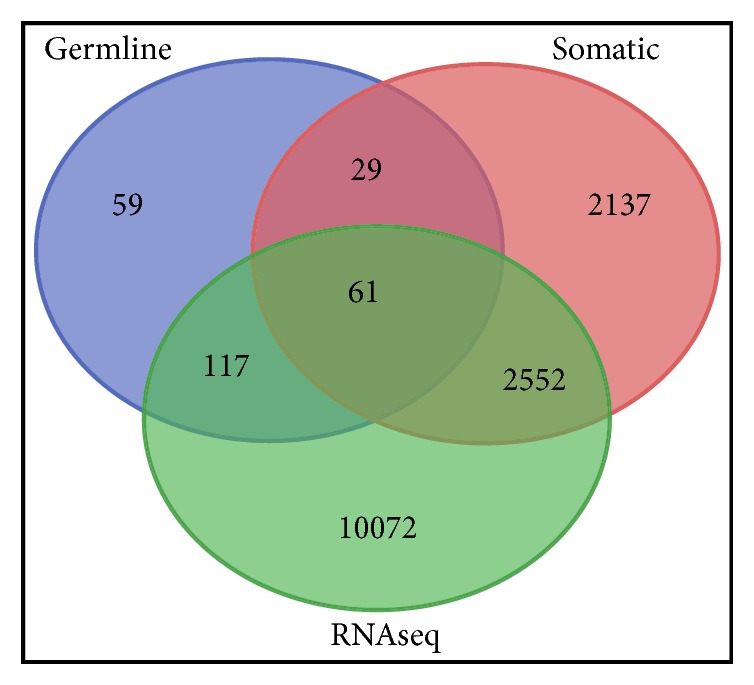
Venn diagram showing differentially and nondifferentially expressed germline and somatic mutated and nonmutated genes. Middle intersections show 61 genes containing both germline and somatic mutations and are also significantly expressed with RNA-seq dataset. Germline indicates genes with germline genetic susceptibility variants from GWAS. Somatic indicates genes with somatic mutations from TCGA. RNA-seq indicates differentially expressed genes evaluated using TCGA gene expression data.

The results of these analyses are presented in a Venn diagram in Figure 2. Out of the 266 genes containing germline mutations evaluated, 90 genes contained both germline and somatic mutations (Figure 2). This analysis also revealed a 61 gene signature containing both germline and somatic mutations. The second investigation produced a 117 gene signature containing germline mutations only (Figure 2). The third investigation yielded a 2552 gene signature containing somatic mutations only (Figure 2). A total of 59 genes contained germline mutations only and were not significantly differentially expressed. A set of 29 genes contained both germline and somatic mutations and were not significantly differentially expressed. Some 2137 genes contained somatic mutations only and were not significantly differentially expressed (Figure 2). About 10072 genes were significantly differentially expressed but contained neither germline nor somatic alterations (Figure 2).

The results showing a signature of the 61 genes containing both germline and somatic mutations are presented in Table 2. The signature includes the genes* PGBD1, OXR1, GZF1, ITGAX, ORC3, BMPR1B, KLK3, KLK2, NKX3-1, SLC22A3, POU5F1B, LMTK2, NAALADL2, LDAH, PDLIM5, SLC22A3, JAZF1, LMTK2, CASC8, DAP2IP, TIMM23B, MSMB, MYEOV, FLT1, SL35B4,* and* HNF1B* containing genetic variants reported to be directly associated with aggressive PCa (Table 2) (references provided in supplementary Table SA). Also presented in the table are the SNP rs-IDs, the GWAS* P* value indicating the strength of germline mutation association PCa, the frequency of germline and somatic mutation events, and the gene expression* P* value indicating the level of significance in expression levels between tumors and control samples for individual genes.

There was significant variation in the distribution of germline and somatic mutations among the genes. Overall the genes containing germline variation did not have a high frequency of somatic mutations (Table 2). The genes* FGFR2, HOXB13, RNASEL, PDLIM5, NKX3-1, KLK2, POU5F1B, KLK3, SLC19A2, SLC22A3, ITGA6, TXB5, ZNF827,* and* SLC41A1* contained more than one germline mutation (Table 2). The genes* KLK3* and* KLK2* were the most highly germline mutated. The genes* LRP1B, BCL11A, SX1, DNAH5, PRDM15, GLI2, TRIM31, TCF7L2, ATF7IP, KIAA1211, ZNF827, FREM1, MDM4, TBX3, LMTK2, STAT3, PKHD1,* and* IL16* contained more than one somatic mutation (Table 2). The gene* LRP1B* was the most highly somatically altered (Table 2). Interestingly, the genes* RNASEL, FTO, BMPR1A, ITGA6, TCF7L2, FREM1, LMTK2,* and* STAT3* containing germline mutations with weak to moderate associations were found to contain germline and somatic mutations and were differentially expressed between PCa and controls (Table 2).

### 3.3. Patterns of Expression Profiles for Containing Germline and Somatic Mutations

**Figure 3:**
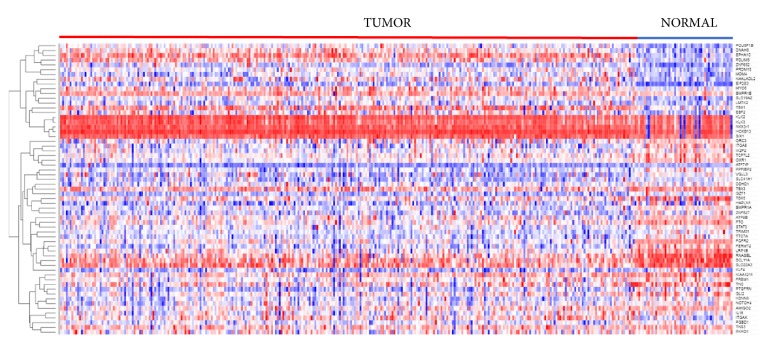
Patterns of expression profiles for the 61 genes containing both germline and somatic mutations distinguishing patients with aggressive tumors and controls generated using hierarchical clustering.

To investigate whether the 61 genes containing both germline and somatic mutations are coregulated and have similar patterns of expression profiles, we performed hierarchical clustering. The results showing the patterns of expression profiles are presented in Figure 3. The analysis revealed that genes containing germline and somatic mutations have similar patterns of expression profiles, suggesting that they are likely to be coregulated and functionally related. As expected, there was significant variation in the patterns of gene expression profiles. The variability in patterns of expression profiles among the genes containing germline and somatic mutations (Figure 3) can partially be explained by the fact that these genes were derived from different populations and different clinical phenotypes. Under such condition the observed outcome is expected. Five genes (*KLK2, KLK3, NKX3-1, HOXB13, and SIX1*) were consistently highly expressed in both tumors and control samples (Figure 3). The genes* EPHA10, PDLIM5, POU5F1B, DNAH5, EIF2S3, SLC19A2, NAALADL2, ZNF652, MYO6, EBF2, TBX1,* and* MD4,* were upregulated in tumors and down regulated in controls (Figure 3).

Additional hierarchical clustering combining the 61 genes containing germline and somatic mutations with the 117 genes containing germline mutations only also revealed similarities in patterns of gene expression profiles (results not presented here). Further analysis combining the 61 genes containing germline and somatic mutations with highly somatic mutated genes (i.e., >5 somatic mutation events per gene) also revealed similarity in patterns of expression profiles (results not presented). This suggests that genes containing germline and somatic mutations are likely coregulated and may not only be involved in* cis* regulation but also in* trans* regulation.

### 3.4. Enrichment Analysis of Molecular Networks and Biological Pathways

To gain insights about the broader biological context through which interactions and cooperation among and between genes containing germline and somatic mutations operate, we performed network and pathway analysis using IPA. We hypothesized that genes containing germline and somatic mutations are functionally related and interact with one another in molecular networks and biological pathways enriched for germline and somatic mutations. Thus, we sought to discover molecular networks and biological pathways enriched for germline and somatic mutations. As a first step, we performed network and pathway analysis including the 61 genes containing germline and somatic mutations from Table 2 and the 34 genes that were found to contain high somatic mutation events (>5 somatic mutation events) from Table 1. In the second step, we combined the 61 genes containing germline variation and somatic mutations and the 34 highly somatic mutated genes with the 117 genes containing only germline mutations. Here we sought to investigate the possible oncogenic interactions between the germline and the somatic genomes. In these analyses, we filtered out genes with spurious and predicted interactions, keeping only genes with ≥ 3 direct connections to ensure the reliability of networks and functional relationships among the genes in the networks.

**Figure 4:**
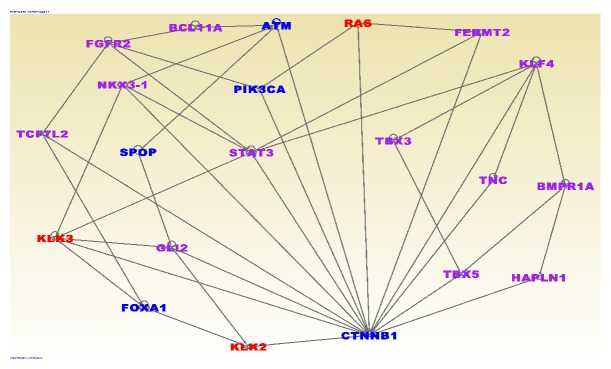
Molecular networks showing interactions among genes containing both germline and somatic mutations. Gene names (symbols) are presented in the nodes. Gene names in purple color font contain both germline and somatic mutations. Gene names in blue color fonts indicate genes with more >5 somatic mutations whereas gene names in red font indicate genes containing germline mutations directly associated with aggressive prostate cancer. Note that the networks were filtered to remove spurious connections.

The results of network and pathway analysis for the 61 genes containing both germline and somatic mutations and the 34 genes with high somatic mutation frequency are presented in Figure 4. In the figure, genes are presented as nodes and the vertices or solid lines indicate relationships among the genes. The font color of the gene symbol indicates the mutation events as described in the figure legend. Interestingly, out of the 95 genes evaluated, 94 were predicted to be significantly involved in cancer (P<1.47x10^−22^), with 68 predicted to be directly involved in PCa.

As expected, the analysis revealed genes with multiple overlapping functions. The network with the highest Z-score (Z-score = 44) contained genes* EPHB1, FERM2, FREM1, GLI2, HOXB13, KIF16B, KLF4, KLK2, KLK3, MDM4, NKX3-1, PCDH18, PKHD1, RNASEL, SIX1, SPOP, TBX1, TBX3, TBX5, *and* VPS13D* predicted to be involved in organism and tissue development. The second networks with a Z-score = 31 revealed the genes* ATF7IP, CTNNB1, EIF2S3, FLG, FOXA1, FZD4, IKZF2, KCNN3, MACF1, PPFIBP2, PRDM15, PTPRD, SLC19A2, STAT3, TCF7L2,* and* TNS3* predicted to be involved in cancer, cellular development, and proliferation.

In the network (Figure 4) the genes in purple color font contain both germline and somatic mutations, the genes in blue color fonts indicate genes with high somatic mutation frequency >5, whereas gene names in red font indicate genes containing germline mutations directly associated with aggressive PCa. As evidenced in Figure 4, the genes containing germline and somatic mutations were found to be functionally related and interacting with one another. In addition, the genes containing germline and somatic mutations were found to be interacting with the most highly somatically mutated genes.

Pathway analysis revealed various signaling pathways enriched for both germline and somatic mutations. Among the most significant pathways included the PCa signaling pathway (P<5.81x10^−6^) with associated genes* ATM, CTNNB1, FGFR2, KLK3, NKX3-1, *and* PIK3CA;* the MSP-RON signaling pathway (P<1.54x10^−5^) with associated genes* ATM, FGFR2, KLK2, KLK3,* and* PIK3CA* and the P53 signaling pathway (P<1.24 x10^−4^) and the associated genes* ATM, CTNNB1, FGFR2, MDM4,* and* PIK3CA*; and the molecular mechanisms of cancer signaling pathway (P<3.19x10^−4^) with associated genes* ATM, BMPR1A, BMPR1B, CTNNB1, FGFR2, FZD4, PIK3CA,* and* PLCB4*. Interestingly, the discovered pathways included genes containing genetic variants directly associated with aggressive PCa such as ATM and KLK3.

**Figure 5:**
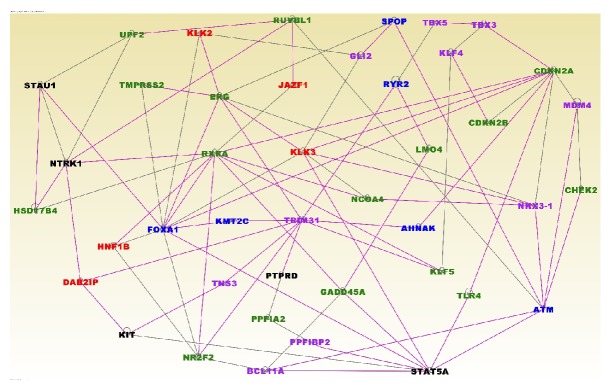
Molecular networks showing interactions among genes containing both germline and somatic mutations. Gene names (symbols) are presented in the nodes. Gene names in purple color font contain both germline and somatic mutations. Gene names in blue color fonts indicate genes with more >5 somatic mutations, gene names in green font indicate genes containing germline mutations only, and gene names in red font indicate genes containing germline mutations directly associated with aggressive prostate cancer. Note that the networks were filtered to remove spurious connections. The colors red and black represent overlap in functional relations among genes in merged networks.

The results of network and pathway analysis based on combining the 61 genes containing germline variation and somatic mutations and the 34 highly somatically mutated genes with the 117 genes containing germline mutations only are presented in Figure 5. Here we sought to investigate the possible oncogenic interactions between the germline and the somatic genomes. The analysis revealed 15 networks containing genes with multiple overlapping functions with z-scores ranging from 2 to 34. The top networks with Z-score 30 – 34 contained genes predicted to be involved in cancer and cell cycle. The results showing the functional relationships among the genes in the three top networks are shown in Figure 5. Network analysis revealed that genes containing germline and somatic mutations, high somatic mutation frequency and genes containing only germline mutations are functionally related (Figure 5). Interestingly, genes containing genetic variants directly associated with aggressive PCa (*KLK2, JAZF1, KLK3,* and* HNF1BP)* were found to be interacting with other sets of genes.

Pathway analysis revealed biological pathways enriched for both germline and somatic mutations. Interestingly, genes containing germline variations only were found to be involved in the same pathways with genes containing somatic mutations only. This suggests that germline and somatic mutations may cooperate through the pathways. The most significant pathways were the MSP-RON signaling pathway (P<3.21x10^−8^), molecular mechanisms of cancer (P<3.31x10^−7^), PDGF signaling (P<4.00x10^−7^), Axonal guidance signaling (P<5.60x10^−7^), P53 signaling (P<1.38x10^−6^) and STAT3 signaling pathway (P<7.40x10^−6^). Interestingly in both network and pathway analysis, genes containing genetic variants with strong and moderate to weak associations were found to be functionally related and interacting with genes containing somatic mutations.

## 4. Discussion

Studies of cancer genomes have been mostly concerned with understanding the somatic mutations or transcriptome changes that arise during tumorigenesis. For many years and decades, germline and somatic mutation information have generally been analyzed and reported as separate endeavors. Hundreds of genetic variants and genes associated with an increased risk of developing PCa have been reported in GWAS [5]. Similarly, hundreds of somatic mutations and genes have been reported [9, 10]. However, emerging evidence indicates that germline variations may interact with somatic events to drive carcinogenesis [33–35]. In this exploratory study, we investigated the possible oncogenic interactions and cooperation between genes containing germline and somatic mutations. We found evidence that genes containing germline mutations also contain somatic mutations, and that these genes are functionally related and interact with one another in molecular networks and biological pathways enriched for both genetic alterations. Our analysis reveals that oncogenic interactions and cooperation between germline and somatic mutations likely occurs through molecular networks and biological pathways. To our knowledge this is the first study to show that genes containing germline mutations also contain somatic mutations and that these genes interact with one another in molecular networks and biological pathways in aggressive PCa. The novel aspect of the study is that germline and somatic mutation may interact and cooperate through molecular networks and biological pathways to drive aggressive PCa. The clinical significance of these results is that such information could be used for the development of new precision prevention strategies and polygenic risk scores to identify men at high risk of developing aggressive PCa [6, 7].

In this study, evaluation of somatic mutation profiles revealed that genes containing genetic susceptibility variants did not exhibit an overall increase in somatic mutation frequency. This is consistent with the results of a recent study which explored somatic mutation profiles within susceptibility regions in a range of cancers [36]. In that report, the authors found that genes in cancer susceptibility regions did not exhibit high somatic mutation frequency [36]. However, the published analysis considered overall somatic mutations, which represented a substantial proportion of passenger mutations, while our study focused on network and pathway analysis to gain insights about the broader biological context in which germline and somatic mutations may cooperate to drive aggressive PCa. Importantly, network and pathway analysis showed that germline and somatic mutations were involved in many common signaling pathways which have been implicated in PCa. Interestingly, many highly somatically mutated genes not containing germline mutations were found to interact with germline mutated genes in biological pathways. This suggests that cooperation between germline and somatic alteration may not only involve* cis* regulation but also* trans* regulation through molecular networks and biological pathways. The clinical significance is that such pathways could serve as targets for the development of novel targeted therapeutics. Although we did not investigate the regulatory mechanisms and the impact of germline variation on gene expression in this study, association of PCa risk variants with gene expression in tumor and normal tissue has been reported [37]. Moreover, cis-regulatory variation in the human prostate transcriptome by using gene-level allele-specific expression has been reported [38].

The low somatic mutation frequency in germline mutated genes could be due to natural selection which is likely to confer selective advantage to specific genes [39]. For example, genomic alteration, clonal selection, and evolution of the tumor microenvironment, may contribute towards unique physiological characteristics under selection pressure contributing to increased mutations in the somatic or tumor genome as observed in this study for the most highly somatic mutated genes, a phenomenon which has been reported in PCa [39]. Although our approach focuses on PCa, the methodology and approach could be applied to other cancers as well.

This exploratory study investigated the possible oncogenic interactions and cooperation among and between genes containing germline and somatic mutations. However, limitations of the study must be acknowledged. Important limitations include the lack of consistent methodological reporting between studies examining low and high grade PCa in GWAS studies which can confound the results. Additionally, genetic variants and genes from GWAS were derived from many diverse populations. Gene expression can be population-specific and there is evidence from the literature that some genetic susceptibility variants may confer population-specific risks [40]. It is worth noting that this study focused on men of European ancestry because the population is well represented in both GWAS and TCGA. Future studies should include other ethnic populations, especially those underrepresented in genomic studies like African American men who are disproportionately affected by aggressive PCa.

## 5. Conclusions

Our findings suggest possible oncogenic interactions and cooperation between genes containing germline variation and somatic mutations. The results suggest a complex interplay between germline and somatic variation, underscoring the need for further studies to understand how the genetic variants and somatic alterations interact to drive aggressive PCa in different populations. Additionally, functional experiments are warranted to uncover the molecular mechanisms underlying these interactions and cooperation between the germline and the somatic genomes.

## Figures and Tables

**Table 1:** List of the 34-gene signature containing the most highly somatically mutated genes (≥ 5 Mutation events) that were significantly differentially expressed between aggressive tumors and controls.

*Genes*	Region	Mutation events	DE *P* value
*SPOP*	17q21.33	29	2.13E-25
*ATM* ^*∗∗*^	11q22.3	13	0.004526
*FOXA1*	14q21.1	12	1.41E-31
*KMT2C*	7q36.1	12	0.04799
*LRP1B*	2q22.1	10	5.18E-31
*FAT3*	11q14.3	9	1.69E-39
*OBSCN*	1q42.13	9	7.66E-05
*FREM2*	13q13.3	8	4.60E-12
*CSMD3*	8q23.3	8	0.000312
*KIF16B*	20p12.1	8	0.020567
*PCDH18*	4q28.3	7	2.00E-15
*SYNE1* ^*∗∗*^	6q25.2	7	1.12E-07
*AHNAK*	11q12.3	7	6.32E-06
*PLCB4*	20p12.3	7	0.000102
*FAT2*	5q33.1	6	2.13E-31
*FAM83B*	6p12.1	6	8.65E-31
*DCHS2*	4q31.3	6	4.08E-27
*CDH23*	10q22.1	6	1.01E-26
*MACF1*	1p34.3	6	9.90E-06
*FLG*	1q21.3	6	2.97E-05
*PIK3CA*	3q26.32	6	3.39E-05
*VPS13D*	1p36.22	6	4.42E-05
*HFM1*	1p22.2	6	0.003362
*PTPRD*	9p24.1	6	0.017381
*EPHB1*	3q22.2	5	9.49E-41
*KIAA1614*	1q25.3	5	6.36E-26
*AHNAK2*	14q32.33	5	8.04E-26
*MXRA5*	Xp22.33	5	2.48E-07
*SACS*	13q12.12	5	7.04E-07
*ASH1L*	1q22	5	6.85E-05
*RYR2* ^*∗∗*^	1q43	5	8.91E-05
*CTNNB1* ^*∗∗*^	3p22.1	5	0.000345
*MT-ND5*	21q22.3	5	0.014434
*FZD4* ^*∗∗*^	11q14.2	5	0.042185

*Note.* DE indicates differential expression. *∗∗* indicates genes associated with aggressive PCa.

**Table 2:** List of the 61-gene signature containing both germline and somatic mutations found to be significantly differentially expressed between aggressive tumors and controls. *∗∗* indicates the gene containing genetic variants directly associated with aggressive PCa.

	GWAS			DE		
*Genes*	Chrom	SNP_ID	*P* value	Germline	Somatic	*P* value
*PGBD1* ^*∗∗*^	6p22.1	rs1233708	0.004	1	2	0.02169
*OXR1* ^*∗∗*^	8q23	rs16901979	0.002	1	1	1.52E-20
*GZF1* ^*∗∗*^	20p11.21	rs6076072	0.01	1	1	2.42E-12
*ITGAX* ^*∗∗*^	16p11.2	rs8045738	0.006	1	1	1.20E-11
*ORC3* ^*∗∗*^	6q15	rs9450716	0.002	1	1	3.31E-06
*BMPR1B* ^*∗∗*^	4q22	rs17021918	0.03	1	1	3.17E-06
*KLK3* ^*∗∗*^	19q13.41	rs2739472	9.00E-186	15	1	4.43E-13
*KLK2* ^*∗∗*^	19q13.33	rs1354774	6.00E-20	5	1	4.53E-19
*NKX3-1* ^*∗∗*^	8p21.2	rs13272392	4.00E-34	4	1	3.30E-20
*SLC22A3* ^*∗∗*^	6q25.3	rs4646284	3.20E+52	4	1	6.62E-11
*RNASEL*	1q25	rs486907	0.004	3	1	8.88E-26
*ZNF827*	4q31.22	rs56935123	4.00E-09	2	3	9.16E-06
*FGFR2*	10q26.12	rs10886902	2.00E-53	2	1	8.24E-29
*HOXB13*	8q24.21	rs188140481	6.00E-34	2	1	2.36E-27
*PDLIM5*	4q22	rs17021918	4.20E-15	2	1	1.36E-21
*POU5F1B* ^*∗∗*^	8q24.21	rs16901979	1.00E-16	2	1	4.94E-17
*SLC19A2*	1q23.3	rs3765227	1.26E-04	2	1	6.46E-11
*ITGA6*	2q31.1	rs126212278	0.001	2	1	5.56E-10
*TBX5*	12q24.1	rs1270884	6.75E-11	2	1	1.53E-09
*SLC41A1*	1q32.1	rs6679073	4.00E-15	2	1	0.026695
*LRP1B*	2q21.2	rs10210358	2.00E-06	1	10	7.28E-33
*PKHD1*	6p21.2	rs10498792	3.00E-06	1	4	0.01337
*ATF7IP*	12p13.1	rs3213764	2.00E-09	1	3	1.13E-06
*FREM1*	9p22.3	rs1552895	0.002	1	3	7.30E-05
*BCL11A*	2p16.1	rs2556375	6.00E-19	1	2	2.02E-17
*SIX1*	14q23.1	rs7153648	2.00E-09	1	2	4.77E-16
*DNAH5*	5p15.2	rs4463179	2.00E-06	1	2	8.71E-16
*PRDM15*	21q22.3	rs6586243	7.79E-06	1	2	6.34E-13
*GLI2*	2q14	rs11122834	5.00E-06	1	2	1.28E-11
*TRIM31*	6p22.1	rs115457135	1.00E-07	1	2	4.61E-11
*TCF7L2*	10q25.3	rs7903146	0.009	1	2	1.43E-08
*KIAA1211*	4q12	rs629242	7.25E-07	1	2	5.48E-06
*MDM4*	1q32	rs4245739	2.01E-11	1	2	9.95E-05
*TBX3*	12q24.21	rs11067228	1.00E-14	1	2	0.000208
*LMTK2* ^*∗∗*^	7q22.1	rs6465657	0.007	1	2	0.00243
*STAT3* ^*∗∗*^	17q21	rs744166	0.03	1	2	0.002775
*IL16*	15q26.3	rs7175701	9.80E-08	1	2	0.026789
*EPHA10*	1p34.3	rs731174	5.00E-06	1	1	1.44E-35
*FERMT2*	14q22.1	rs8008270	1.78E-14	1	1	3.20E-30
*FTO*	16q12.2	rs9939609	0.04	1	1	6.96E-16
*BMPR1A*	10q22.3	rs11597689	0.03	1	1	1.09E-13
*EIF2S3*	Xp22.11	rs6627995	1.00E-13	1	1	1.21E-12
*TTC7A*	2p16.3	rs10194115	5.00E-07	1	1	1.81E-11
*NAALADL2* ^*∗∗*^	3q26.31	rs78943174	4.00E-08	1	1	4.07E-10
*PPFIBP2*	11p15.4	rs12791447	4.00E-08	1	1	5.20E-10
*KCNN3*	1q21.3	rs1218582	1.95E-08	1	1	9.96E-10
*AMIGO2*	12q13.11	rs2711721	2.00E-06	1	1	2.17E-09
*ZNF652*	17q21.32	rs7210100	3.40E-13	1	1	5.55E-09
*MYO6*	6q14.1	rs9443189	4.00E-08	1	1	2.71E-08
*EBF2*	8p21.2	rs11135910	8.00E-11	1	1	8.98E-08
*TBX1*	22q11.21	rs2238776	2.00E-08	1	1	7.97E-08
*PTGFRN*	1p13.1	rs2806864	6.00E-07	1	1	2.52E-07
*TNC*	9q33.1	rs7847271	4.00E-06	1	1	3.93E-07
*ATP9B*	18q23	rs7241993	2.00E-09	1	1	4.37E-06
*HAPLN1*	5q14.3	rs4466137	3.00E-06	1	1	6.25E-05
*NOTCH4*	6p21.3	rs3096702	4.78E-09	1	1	5.81E-05
*VGLL3*	3p12.1	rs9757252	5.00E-06	1	1	7.72E-05
*IKZF2*	2q13.1	rs7569918	8.85E-04	1	1	9.51E-05
*KLF4*	9q31.2	rs817826	5.00E-14	1	1	0.002599
*TNS3*	7p12.3	rs56232506	9.00E-09	1	1	0.007265
*DDHD1*	14q22.1	rs8008270	2.00E-14	1	1	0.008911

*Note.* DE indicates differential expression.

## Data Availability

Germline mutation information from GWAS along with sources of original information is provided in the supplementary materials in supplementary Table SA. Original data on somatic mutations, gene expression data, and clinical information is available at the TCGA via the Genomics Data Commons https://gdc.cancer.gov/.

## Supplementary Materials

Supplementary Materials Table S1B: list of significantly differentially expressed nonsomatic mutated genes associated with aggressive PCa along with estimates of* P* values, adjusted* P* values, and log fold change. Table SA: a comprehensive list of single nucleotide polymorphisms (herein called genetic variants). Table S1A: list of significantly differentially expressed somatic mutated genes associated with aggressive PCa.
